# Electronic Problem-Solving Treatment: Description and Pilot Study of an Interactive Media Treatment for Depression

**DOI:** 10.2196/resprot.1925

**Published:** 2012-09-25

**Authors:** James Albert Cartreine, Steven E Locke, Jay C Buckey, Luis Sandoval, Mark T Hegel

**Affiliations:** 1 Program on Behavioral Informatics and eHealthDepartment of PsychiatryBrigham and Women's HospitalBoston, MAUnited States; 2 Harvard Medical SchoolBoston, MAUnited States; 3 Department of PsychiatryMassachusetts General HospitalBoston, MAUnited States; 4 Department of PsychiatryBeth Israel Deaconess Medical CenterBoston, MAUnited States; 5 The Undersea and Space Medicine Research LabDepartment of MedicineThe Geisel School of Medicine at DartmouthLebanon, NHUnited States; 6 Department of Educational PsychologyUniversity of TexasAustin, TXUnited States; 7 Division of Behavioral MedicineDepartment of PsychiatryThe Geisel School of Medicine at DartmouthLebanon, NHUnited States

**Keywords:** Depression, problem-solving therapy, computer-based therapy, interactive media, Internet intervention, problem-solving treatment, cognitive behavioral therapy

## Abstract

**Background:**

Computer-automated depression interventions rely heavily on users reading text to receive the intervention. However, text-delivered interventions place a burden on persons with depression and convey only verbal content.

**Objective:**

The primary aim of this project was to develop a computer-automated treatment for depression that is delivered via interactive media technology. By using branching video and audio, the program simulates the experience of being in therapy with a master clinician who provides six sessions of problem-solving therapy. A secondary objective was to conduct a pilot study of the program’s usability, acceptability, and credibility, and to obtain an initial estimate of its efficacy.

**Methods:**

The program was produced in a professional multimedia production facility and incorporates video, audio, graphics, animation, and text. Failure analyses of patient data are conducted across sessions and across problems to identify ways to help the user improve his or her problem solving. A pilot study was conducted with persons who had minor depression. An experimental group (n = 7) used the program while a waitlist control group (n = 7) was provided with no treatment for 6 weeks.

**Results:**

All of the experimental group participants completed the trial, whereas 1 from the control was lost to follow-up. Experimental group participants rated the program high on usability, acceptability, and credibility. The study was not powered to detect clinical improvement, although these pilot data are encouraging.

**Conclusions:**

Although the study was not powered to detect treatment effects, participants did find the program highly usable, acceptable, and credible. This suggests that the highly interactive and immersive nature of the program is beneficial. Further clinical trials are warranted.

**Trial Registration:**

ClinicalTrials.gov NCT00906581; http://clinicaltrials.gov/ct2/show/NCT00906581 (Archived by WebCite at http://www.webcitation.org/6A5Ni5HUp)

## Introduction

Until now, one commonality among computer-automated depression interventions has been a heavy reliance on users reading text to receive the intervention. However, these interventions have not fully exploited the capacity of computers to deliver interactive media [1] to guide treatment. However, text-delivered interventions place a burden on persons with depression because these users may lack the literacy, concentration, or energy or motivation needed to read large quantities of text. Moreover, text conveys only verbal content, not the nonverbal cues (ie, body language and prosody) that an empathic therapist would use. Text alone cannot simulate the psychotherapy experience with high fidelity.

Programs can be designed to respond to users’ inputs by playing video and audio clips, based on branching algorithms, to guide users through evidence-based treatments. Such programs could deliver some of the nonspecific aspects of therapy by conveying an on-camera therapist’s warmth, personality, compassion, and ability to remain supportive when the patient experiences setbacks [2].

The purpose of this study was to explore the possibility of using interactive media—particularly video—to deliver computer-automated treatment for depression. This paper describes an interactive multimedia program that provides an automated electronic version of problem-solving therapy (*ePST*) for depression and is designed to simulate the experience of being in treatment with a master clinician. It also reports a pilot study that was conducted with 14 persons with minor depression.

### Problem-Solving Treatment

The *ePST *program is directly based on a manualized, evidence-based treatment for depression known as problem-solving treatment for primary care (PST-PC). PST-PC has been demonstrated to be effective for the treatment of depression [3-5]. (For a detailed description of PST-PC, see Hegel and Arean [6].) The basis of PST-PC is that enhancing problem-solving skills and attitudes and working to solve problems in one’s life can reduce depression. PST-PC involves 6 steps: (1) clarifying the problem, (2) establishing an achievable goal, (3) brainstorming alternative solutions, (4) evaluating the pros and cons of each solution and selecting one or more solutions, (5) developing an action plan to implement the solution(s), and (6) evaluating the success of the implementation and troubleshooting as needed. Each session concludes with scheduling pleasant activities.

The mechanism by which problem-solving treatment works has not yet been clearly identified [7]. PST-PC activates individuals to take steps toward changing their situation. Evidence is emerging to suggest that, along with typical predictors of improvement such as treatment adherence [8], the most important element of PST-PC for overcoming depression may be helping the patient to overcome an avoidant coping style [7,9]. Because PST-PC has strong face validity and is easy for most patients to understand, acceptance and satisfaction from patients is high, with very low dropout rates, as reported in clinical trials of PST-PC [5,10].

### The ePST Program

### Purpose

An interactive media program was produced to deliver *ePST*. The goal of *ePST *was to automate an empirically supported treatment and to provide it in the context of a simulated helping relationship. The *ePST *program was built for the US National Aeronautics and Space Administration (NASA) as part of a suite of self-guided programs to help astronauts manage their own psychosocial problems on long missions [11]. The system is used autonomously, confidentially, and in a self-directed manner. Although the development of *ePST *was funded by NASA, it was designed to also be evaluated among the public with a wide variety of patients. Therefore, a NASA and a general public version were produced, which differ only by a few video clips. As such, *ePST *is intended for use by both astronauts and nonastronauts.

### Theoretical Underpinning

*ePST *is based on the Virtual Practicum Model [12,13], an approach to designing professional education programs, which is based on Boisot’s “epistemological space” [14], Kolb’s “learning cycles” [15], and Schon’s “reflective practicums” [16]. The Virtual Practicum Model has been used to teach clinicians how to conduct counseling in prevention of human immunodeficiency virus infection [17], manage patients with human immunodeficiency virus/acquired immunodeficiency syndrome [18], and conduct genetic counseling and testing [19]. The basis of the Virtual Practicum Model is the simulation of the interactive experience of being trained one-on-one by a master clinician—a practitioner who is both a master of his or her specialty and a master teacher. The goal of the program is to provide trainees both the concrete information they need to manage clinical cases and to impart the soft skills needed to interact effectively with patients, and the case conceptualization skills needed to understand each unique case. *ePST *builds on the Virtual Practicum Model by providing persons seeking treatment for depression an opportunity to receive treatment (in a simulated fashion) from a master clinician (author MTH) who is an expert in problem-solving treatment. However, not only does the on-camera clinician walk the user through the concrete steps of problem-solving treatment, he conveys warmth and empathy—the soft skills of a skilled psychotherapist. The goal of both the VPM and *ePST *is for users to receive training or treatment from an expert in the field, and thereby to increase the number of persons who can benefit from that professional.

### Program Walk-Through

**Figure 1:**
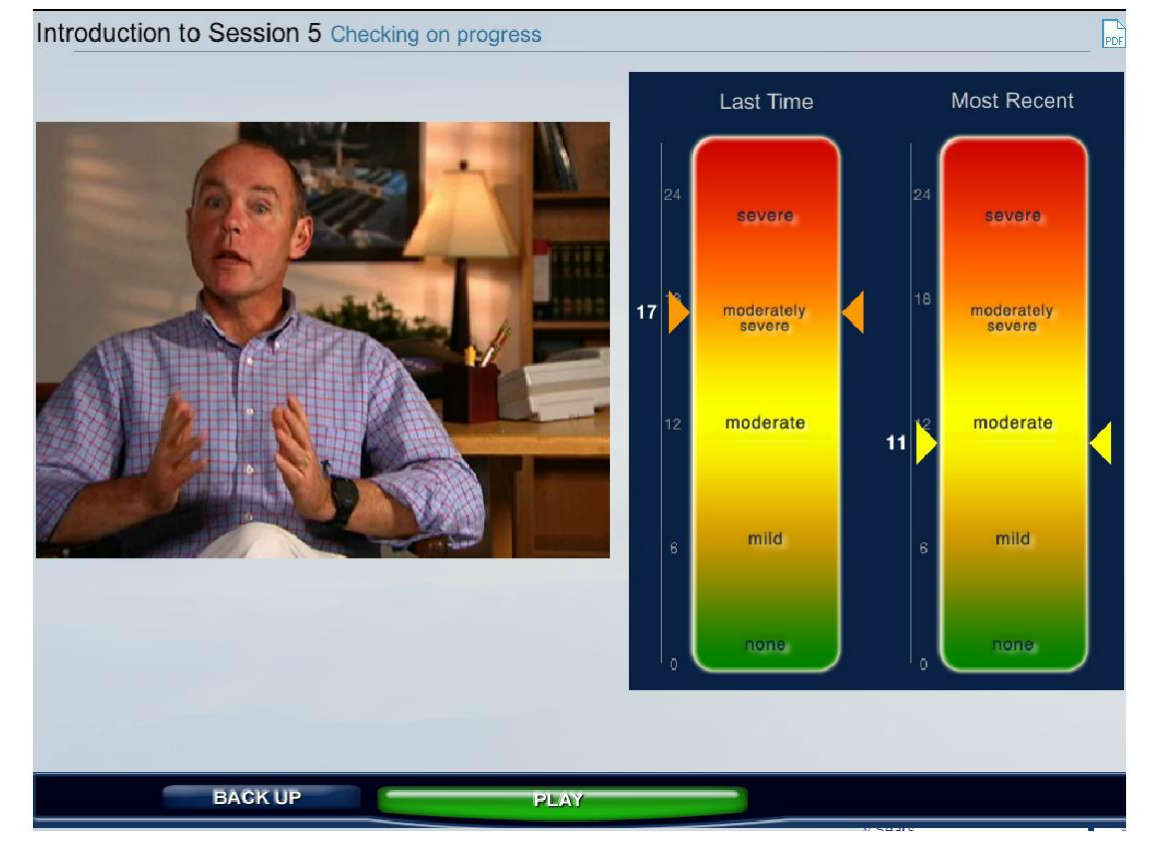
Virtual therapist discusses the Patient Health Questionnaire-9 (PHQ-9) results with the user. Information is provided both graphically and on video.

*ePST *is a multimedia-based, video-intensive program that simulates therapy based on the PST-PC treatment manual [6]. It includes six sessions, intended to be completed once per week. In each session, users are welcomed by a therapist (author MTH) presented via audio and video, who appears in a warmly lit professional office. The user then completes the Patient Health Questionnaire-9 (PHQ-9) [20] measure of depression. Tailored feedback about his or her progress is provided by the virtual therapist, discussing the user’s current level of depression and change from the previous session (see Figure 1).

The first session provides psychoeducation about depression and the process of problem-solving treatment; subsequent sessions offer decreasing guidance on the process of problem-solving treatment. During each session, the virtual therapist guides users through all steps of problem-solving treatment, plus making a list of enjoyable activities (entering their own or choosing from an extensive list of common activities) and scheduling them for the week.

Sessions 2 through 6 begin with a check-in on all active problems. The therapist provides feedback about the user’s success or failure in solving problems compared with the previous session and helps him or her identify ways to improve problem solving, both on individual problems and in general.

Although the process of problem solving remains consistent throughout the program, a variety of audio and video clips are used to maintain a sense of novelty in each session and to respond to user inputs. At the end of the treatment session, the program produces a printout that summarizes the work the user has done on each problem, including his or her action plans, plus a day-by-day schedule of enjoyable activities for the coming week.

The program’s interfaces are designed for ease of use: where free text entry is requested, no other options are available to the user; where menus are used, five or fewer choices are generally made available. Nonetheless, in the background a highly complex program is running, with substantial branching of video and audio, failure analysis algorithms, and data handling methods that adapt to the user’s clinical status and problem-solving history within *ePST*.

Fogg and colleagues have identified factors that engender users’ trust in software or websites and found that the credibility depends largely on three factors: (1) the usability of the program (especially that it does what the user expects it to do at any given moment), (2) the brand or source behind it, and (3) the professionalism of the interface and media [21,22]. If a program behaves in an unpredictable manner, is presented by an unknown entity, or looks amateurish, users are unlikely to trust it. An effort was made to enhance the credibility of *ePST *by addressing these three domains: (1) the program was designed to be simple to use and went through initial usability testing by persons who had been treated for depression, (2) familiar brands are referenced in the program (Harvard, NASA, Dartmouth), and (3) the video and audio for *ePST *were produced in a broadcast television facility, meeting industry standards for production values, and *ePST*’s interfaces were developed by professional graphic artists.

The initial version of the program was delivered via universal serial bus flash drive, which is a convenient format for astronauts, since they often train in settings without Internet access, and bandwidth on space missions is limited. However, *ePST *can readily be adapted for delivery via the Internet.

### Unique Characteristics

*ePST *advances the field of computer-automated interventions for depression by (1) approximating actual treatment via the use of rich media, (2) minimizing the amount of text that a user needs to read, and (3) performing failure analyses to assist users to improve their problem-solving effectiveness.

### Rich Media and the Approximation of Therapy in ePST

Information richness refers to “the potential information carrying capacity of data” [23]. Media can be ranked on richness by the number of channels of communication they employ: verbal (the words said), paraverbal (how they are said), and nonverbal (body language) communication [24]. Although some information is best conveyed by text (eg, procedures or numerical data), emotionally laden information is more effectively communicated with richer media [25]. Psychotherapy falls into the latter category. Through rich media (branching video and audio clips tailored to users’ inputs), a conversation is approximated between the user and a competent, caring therapist. The intent is to make the *ePST *program feel more like interacting with a person than with a computer. *ePST *contains 148 video and 225 audio clips, although each user receives only a fraction of them, based on his or her clinical status and what he or she does in the program.

### Minimization of Text in ePST

Although *ePST *was created for one of the most highly educated groups of individuals in the world—astronauts—it requires less reading skill than most self-help programs, due to the extensive use of audio and video. Text on the screen is written at a low literacy level, mainly limited to labels and occasional short sentences. The user does need to be able to input free text to write his or her own problem statement, goals, and action plan; however, proper grammar and spelling are not required. The writing skill necessary is comparable to that required for sending text messages or emails.

### Failure Analyses in ePST

In in-person problem-solving treatment, a therapist guides the client and provides feedback on how he or she could improve on problem solving. However, without a therapist, the challenge of *ePST *is to guide users through the process of problem-solving treatment while simultaneously teaching them how to evaluate and improve their own work. Failure analysis is a process of examining failures, identifying reasons for them, and planning how to prevent them in the future. It is used in engineering [26], business [27], and software design [28,29]. Through failure analysis algorithms in *ePST*, the computer assists users to identify ways to improve their problem solving in general and where to revise work on specific problems, much as a live therapist would do. In this way, *ePST *tailors each session to what the user has done in the program, thus far.

### Pilot Study Research Questions

The primary purpose of the pilot study was to test research methods and collect preliminary data on which to base a larger clinical trial of *ePST*. As such, we posed the following research questions, noting that the number of participants would likely not be sufficient to test hypotheses: (1) To what extent do participants find *ePST *to be usable? (2) To what extent do participants find *ePST *to be acceptable and credible? and (3) To what extent do participants who use *ePST * improve in depression, compared with those who do not use it?

## Methods

We conducted a feasibility study to establish the methods to be used in a future clinical trial and to elicit initial reactions to the program. The study was conducted at the General Clinical Research Center at Beth Israel Deaconess Medical Center in Boston, MA, USA, and was approved by its institutional review board.

### Participants

Because there are fewer than 100 astronauts [30] (and most do not have depression at any given point), it is necessary to study persons who bear some demographic similarities to astronauts. To support generalization from the test population to astronauts, each participant was between the ages of 30 and 60 years, had completed at least 4 years of college, used a computer at least twice per week, and read and spoke English well enough to use the program. We enrolled only persons meeting the *Diagnostic and Statistical Manual of Mental Disorders*, 4th edition, text revision (DSM-IV-TR) [31] criteria for minor depression and scoring higher than 10 (moderate or worse depressive symptom severity) on the self-report 17-item Hamilton Depression Inventory (HDI) [32].

Exclusion criteria were a history of bipolar disorder or schizophrenia, current substance abuse or dependence, dysthymic disorder, or neurological disorders. Study participants with active suicidal ideation, self-injurious behavior, or previous suicide attempts were also excluded.

### Procedures

We sent 500 email messages to persons listed on a registry of those interested in receiving information about clinical trials for depression. Of these, 104 persons responded. A total of 54 phone screening interviews (to establish age, education, computer experience, and exclusionary diagnoses obtained by history) were successfully completed. Others did not respond to messages. Of the 54 persons screened, we excluded 40 (26 lacked the required education level, 5 reported suicidal ideation or attempts, 4 were excluded for other psychiatric or medical conditions, and 5 were unavailable during the study timeframe). All persons excluded from the study were provided a list of referrals for treatment. This left 14 individuals, who were invited for in-person evaluation. All 14 met eligibility criteria and were enrolled in the study. We randomly assigned 7 participants to each condition.

We conducted the Mini-International Neuropsychiatric Interview (MINI) [33] to establish enrollment eligibility. After enrollment, a baseline assessment was administered and participants were assigned to the waitlist control or *ePST *experimental groups using block randomization to ensure equal numbers of participants in each group. Because the number of available participants was small, we made no attempt to balance groups on any variable.

Participants in the *ePST *condition were requested to use the program weekly on site at the clinic, although they were permitted to schedule sessions less frequently. However, we decided a priori that a longer than 3-week interval between sessions would be considered a protocol violation. We contacted persons in the waitlist (control) group 3 weeks after enrollment to assess depression and safety. A follow-up assessment of depression was conducted at week 6 for control group participants and at posttreatment 1 week after the final *ePST *session (nominally at 7 weeks) for *ePST *group participants. We conducted an additional follow-up with those in the *ePST *group 4 weeks after they completed *ePST*. Participants received payment based on the number of times they were required to visit the clinic: nine times for the *ePST *participants for a total of US $470 and twice for the waitlist participants for a total of US $170. Following the week 6 assessment, waitlist participants were offered off-study use of the *ePST *program.

### Measures

#### Mini-International Neuropsychiatric Interview

The MINI is a short structured diagnostic interview for DSM-IV-TR psychiatric disorders [33]. The MINI demonstrates strong diagnostic concordance with the structured clinical interview for the *Diagnostic and Statistical Manual of Mental Disorders*, 3rd edition, revised, diagnosis of major depression, with Cohen kappa of 0.84, and sensitivity (0.96), specificity (0.88), positive predictive value (0.87), and negative predictive value (0.97) likewise strong [33]. We used the MINI in the enrollment interview only.

#### Hamilton Depression Inventory-17 Item

The HDI [34] is a self-report measure of 17 depressive symptoms used to assess depressive symptom severity over the previous 2 weeks. It emulates the 17-item clinician-administered Hamilton Depression Rating Scale (HDRS) [35]. The response format for individual items varies in scoring from 0 to 2 or 0 to 4 regarding frequency and severity of symptoms, and a total score algorithm is modeled after the HDRS. Scores range from 0 to 53. Consistent with the HDRS, in the HDI scores less than 7 are considered normal, 8-13 indicate mild depression, 14-18 indicate moderate depression, and scores above 18 indicate severe depression.

The HDI shows strong internal consistency (coefficient alpha = .91), strong test–retest reliability (*r *= .96), and strong convergent validity with the clinician-administered HDRS (*r *= .95) and the self-administered Beck Depression Inventory (developed by Beck and colleagues in 1961) (*r *= .92). Factor analysis showed an expected loading of the majority of items on one factor characterized as “depressed mood-demoralization,” which accounted for 43% of the variance in the measure [34].

#### System Usability Scale

The System Usability Scale (SUS) is a 10-item self-report measure of the ease of using computer programs [36,37]. Items are scored on a 5-point scale (0-4) on the strength of agreement with each of 10 statements (eg, “I found the system unnecessarily complex,” “I felt very confident using the program”). Cronbach alpha for interitem agreement is a robust .91. Factor analysis shows only one significant factor, suggesting that the overall score is the best measure of usability. The sum of the individual items (range 0-40) is multiplied by 2.5 to obtain the total score, ranging from 0 to 100, with higher scores indicating better usability. The scale was administered to *ePST *participants after session 1 and session 6 of *ePST*.

#### Credibility Questionnaire

The Credibility Questionnaire (Cred-Q) is a 9-item survey created for this study from several sources. It comprises questions from the research of Fogg and colleagues [22,38] used to assess the credibility of computer programs in general, plus questions about psychotherapy credibility developed by Borkovec and Nau [39]. It also includes 2 items from an in-person problem-solving treatment acceptability study by Thornett and Mynors-Wallis [40]. Examples from the scale are “How much do you believe what the program tells you?” and “How much do you trust the program to help you?”, to which the user responds on a 10-point scale (1 = not at all; 10 = completely). The Cred-Q was administered at week 7 to the *ePST *participants.

#### Assessment of Self-Guided Treatment

The Assessment of Self-Guided Treatment (AST) is an unpublished measure (written personal communication with C Zayfert, PhD, The Geisel School of Medicine at Dartmouth, August 2003) that we adapted for this study to assess the acceptability of the *ePST *self-guided treatment program. This instrument included 16 statements (eg, “Doing problem-solving treatment using this program was acceptable to me,” “I would feel comfortable using this program without a clinician’s supervision”) that the user responded to on a 7-point scale, which ranged from 1 (strongly disagree) to 7 (strongly agree).

Participants completed the HDI before using the *ePST *program and at 1- and 4-week follow-ups after completing *ePST*. The SUS was completed after sessions 1 and 6, the Cred-Q after session 5, and the AST after session 4.

### Analyses

We recruited a sample of 14 participants, 7 per group. Because the primary aims of this evaluation were to gauge the usability and acceptability of *ePST *and refine the evaluation procedures, the number of participants was based on resources available, not on a power analysis. For the pilot study, due to the small sample size, nonparametric statistical analyses were conducted on all measures. These included the Wilcoxon signed rank test and the Mann-Whitney *U *test. Cronbach alpha was calculated for Cred-Q and AST. The numerical values are presented with mean (SD) or median (range), or both. We used the Statistical Analysis Software (SAS) program, version 9.3 (SAS Institute, Cary, NC, USA) for the analysis.

## Results

### Participants and Group Equivalence

**Figure 2:**
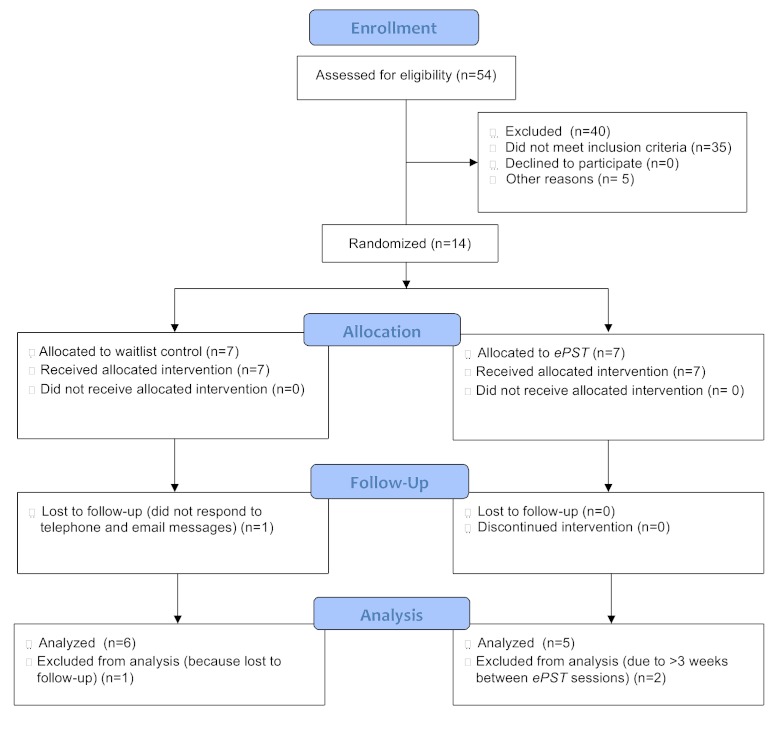
Consort diagram.

As Figure 2 shows, 54 persons responded to an advertisement for the study and were screened by telephone. A total of 35 of them did not meet all inclusion criteria and 5 were excluded for other reasons; 14 participants were enrolled.

Mean HDI scores for the *ePST *group at pretreatment were 15.61 (SD 9.64), and the median score was 15.4 (range 3.2–31.1). For the waitlist control group, the mean HDI score was 18.71 (SD 6.65) and the median was also 15.4 (range 11.4–26). A Mann-Whitney *U *test indicated no significant difference in HDI scores between the groups (*P *= .62) at baseline. We did note demographic differences between the groups (see Table 1).

**Table 1:** Participant demographics.

Characteristic		Waitlist (n = 7)	*ePST* ^a ^(n = 7)	
Age (years), mean (SD)		52.1 (7.7)	48.6 (10.2)
Female, n (%)		5 (71%)	5 (71%)
**Race, n**			
	White	6	7
	Black	1	0
**Ethnicity, n**			
	Hispanic	2	0
	Other	5	7

^a ^Electronic problem-solving treatment.

### Treatment Completion

One participant in the control group was lost to follow-up. All *ePST *group participants completed all *ePST *sessions and posttreatment evaluations; however, 2 persons had a gap of greater than 3 weeks between two sessions, constituting a protocol violation. They were permitted to continue using *ePST*, and we conducted separate analyses on the intent-to-treat sample (n = 13) and the subset completing treatment according to the protocol (n = 11). The mean time to complete each session was 53 (SD 31) minutes, with earlier sessions running longer than later ones.

### Usability, acceptability, and Credibility

The *ePST *group completed the SUS after session 1 and session 6. On the 100-point SUS, the mean score was 80.36 (SD 19.28) and median was 85 (range 42.5–97.5) after session 1. After session 6, the mean SUS was 85.36 (SD 15.91) and median was 95 (range 57.5–100). No significant difference was found for the SUS between the two time points (*P *< .09; Wilcoxon signed rank test).

Cronbach alpha was .922 for the Cred-Q and .830 for the AST. Nonetheless, since the Cred-Q was created for this study and since data have not been previously published on the AST, an item-level analysis of those questionnaires is more appropriate than summary data. Mean and median scores for each Cred-Q item are presented in Table 2; AST scores are presented in Table 3.

**Table 2:** Credibility Questionnaire scores (n = 7).

Credibility item	Mean (SD)	Median (range)
How much do you believe what the program tells you?	7.71 (1.89)	8 (4–10)
How much do you trust the program to help you?	7.43 (2.23)	8 (3–10)
How competent is the program at treating depression?	7.43 (1.90)	8 (4–10)
How credible is the program?	7.71 (2.50)	8 (3–10)
How unbiased is the program?	7.57 (2.82)	8 (2–10)
How expert is the program?	7.86 (1.35)	8 (6–10)
How logical is the treatment?	8.43 (1.27)	8 (7–10)
How much do you think your depression got better?	7.14 (2.34)	8 (3–10)
How much would you recommend the treatment program to a family member or a friend?	7.86 (2.54)	8 (3–10)

**Table 3:** Acceptability of Self-guided Treatment Questionnaire scores (n = 7).

Item	Mean (SD)	Median (range)
I felt comfortable using the computer	6.00 (1.53)	7 (3–7)
Doing problem-solving treatment using this program was acceptable to me	6.29 (1.11)	7 (4–7)
Using the program helped me to do problem-solving treatment	6.14 (1.07)	6 (4–7)
I would rather do problem-solving treatment with a therapist than with the computer	5.00 (1.41)	4 (4–7)
I would rather use a computer to help myself privately than go to a therapist	5.00 (0.82)	5 (4–6)
Computer programs can help people with emotional problems, such as depression	5.57 (1.72)	6 (2–7)
I would feel comfortable using this program without a clinician’s supervision	6.43 (0.79)	7 (5–7)
I felt safe using the program to do problem-solving treatment	5.29 (1.38)	5 (4–7)
I would feel safe doing self-guided treatment for depression on my own without a clinician’s supervision	6.00 (1.00)	6 (5–7)
I would recommend this program to a friend who was also in need of treatment for depression	5.71 (0.76)	6 (5–7)
Using the program helped me to feel better	6.00 (1.29)	7 (4–7)
I believe I would feel comfortable using the program at home on my own computer	5.86 (0.90)	6 (5–7)
I felt comfortable answering questions about my depression symptoms using this program	4.14 (0.69)	4 (3–5)
Using the program helped me to cope with my depression in real life	4.86 (0.38)	5 (4–5)
I felt comfortable using this program without a clinician’s assistance	5.86 (1.07)	6 (4–7)
Following the program’s guidelines for in-between session tasks and homework was acceptable to me	6.14 (0.69)	6 (5–7)

### Effects on Depression

**Figure 3:**
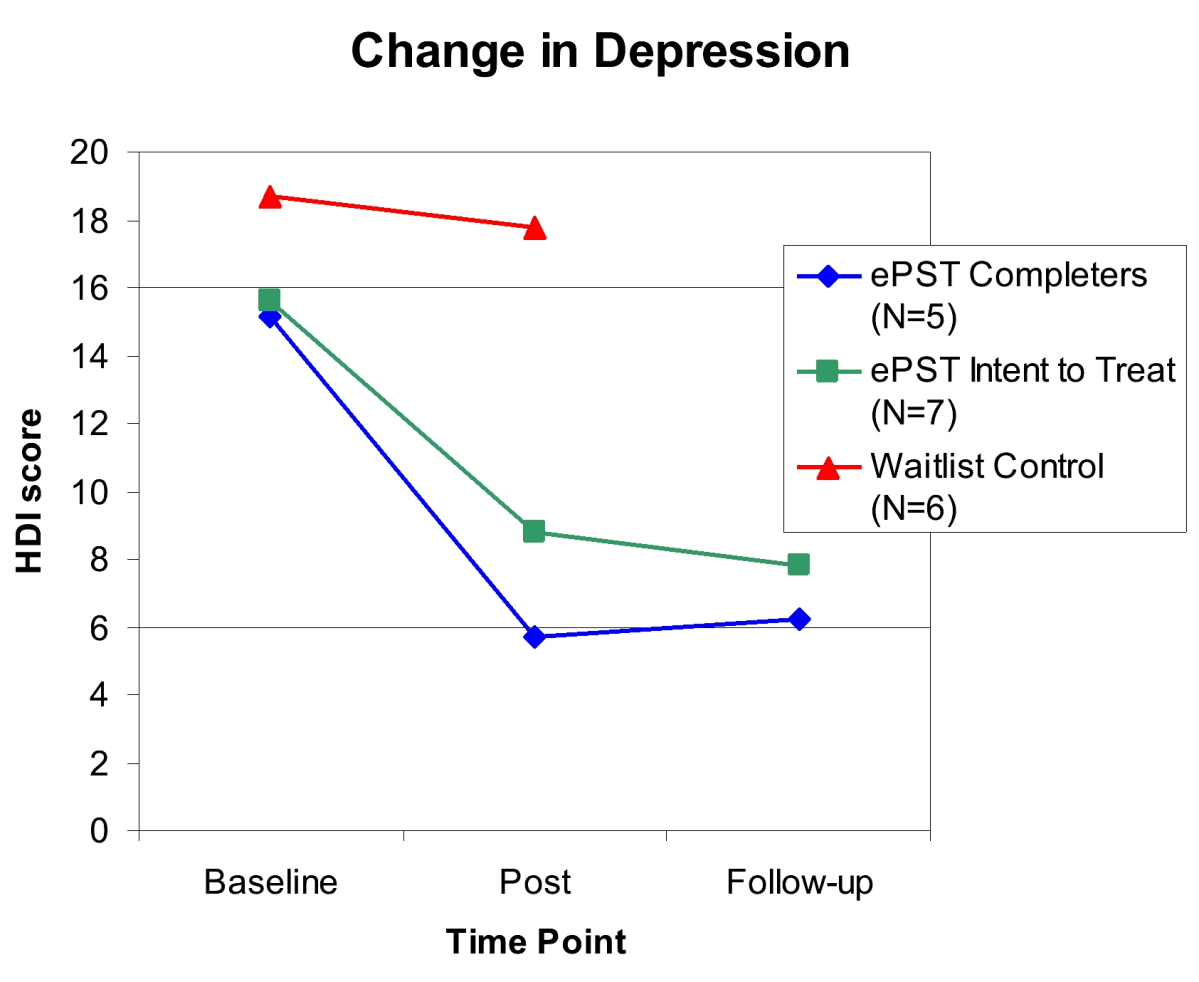
Change in depression.

At posttreatment, in the intent-to-treat analysis (n = 7), the mean HDI score for persons who received *ePST *was 8.81 (SD 5.91) and median was 6.3 (range 1.1–16.7) at posttreatment. For the *ePST *group, the mean percentage change in HDI score from baseline to posttreatment was –35.86% (SD 40.16%) (median –51.02%, range –33.87% to 79.74%). The mean HDI score for the waitlist control group at 6-week follow-up was 17.78 (SD 7.52) (median 18.55, range 8.2–28.4). The mean percentage change in depression for the control group was –11.12% (SD 18.70%) (median –17.78%, range 14.29%–28.07%; see Table 4). The difference in percentage change in depression between the two groups, using the Mann-Whitney *U *test, was not significant in the intent-to-treat analysis (*P *= .25). A Wilcoxon signed rank test indicated that depression scores remained stable at 4-week follow-up for the *ePST *group (*P *= .90), as depicted in Figure 3.

Persons who completed treatment with no more than a 3-week gap between any sessions showed a trend toward greater improvement in depression. For this subset of the *ePST *treatment group, depression decreased by an average of 52.65% (SD 28.28%) (median –61.35%, range –79.74% to –5.56%; *P *=.05). There was no significant difference in depression level between 1-week and 4-week follow-ups in the completer analysis (mean 6.24, SD 6.51; median 4.1, range 0.3–12.4). A Wilcoxon signed rank test indicated that depression scores remained stable at 4-week follow-up for the *ePST *group (*P *= .90), as depicted in Figure 3.

**Table 4:** Percentage change in depression as measured by the Hamilton Depression Inventory (HDI).

Group		Baseline HDI		Posttest HDI		Percentage change, baseline to posttest	
		Mean (SD)	Median (range)	Mean (SD)	Median (range)	Mean (SD)	Median (range)
**ePST ^a^**							
	*I*ntent-to-treat (n = 7)	15.61 (9.64)	15.4 (3.2–31.1)	8.81 (5.91)	6.3 (1.1–16.7)	–35.86% (40.16)	–51.02% (33.87 to –79.74)
	*E*fficacy subsample (n = 5)	15.12 (11.33)	16.3 (3.2–31.1)	5.68 (3.06)	6.3 (1.1–9.6)	–52.65% (28.28)	–61.35% (–5.56 to –79.74)
Waitlist control (n = 7)		18.71 (6.65)	15.4 (11.4–26)	17.78 (7.52)	18.55 (8.2–28.4)	–11.12% (18.70)	–17.8% (14.29 to –28.07)	

^a ^Electronic problem-solving treatment.

## Discussion

We developed an interactive multimedia computer program to provide problem-solving treatment for depression. The program is entirely automated and does not require the involvement of a live clinician. Nonetheless, *ePST *is designed to simulate the therapy experience and to feel more like interacting with a person than with a computer. These efforts appear to have paid off, with high scores for usability, credibility, and acceptability in the pilot study. Although efficacy remains to be tested in larger studies, *ePST *appears to be a promising approach to the treatment of depression.

*Usability *describes the ease of use of software or other technology. In a review of 206 studies that used the SUS, the average usability score was 70.14 (SD 21.70) [37], with the upper quartile ranging from 78.51 to 93.93. The mean usability scores in the present study were 80.4 after session 1 and 85.4 after session 6, which suggests that the program is easy to use from the outset and remains so.

 Ratings on the Cred-Q suggest that participants found the program to be credible for treating their depression. In comparison, Thornett and Mynors-Wallis [40] conducted a study of problem-solving treatment and asked two of the same questions: (1) “How logical is the treatment?” and (2) “How much would you recommend the treatment to a family member or a friend?” On the 10-point scale, the median ratings for those treated by a nurse were 8 and 8, respectively, and for those treated by the general practitioner were 8 and 7, respectively. The median ratings on those questions for *ePST *were 8 and 9, respectively, which suggests similar credibility for the *ePST *program and live clinicians; however, this comparison needs to be further explored in future clinical trials.

Responses to the AST suggest that persons with depression find *ePST *to be acceptable. Of particular note were answers (on a scale of 1 to 7; strongly disagree to strongly agree) to the items “Doing problem-solving treatment using this program was acceptable to me” (mean 6.3, SD 1.1; median 7, range 4–7) and “I would feel comfortable using this program without a clinician’s supervision” (mean 6.4, SD 0.8; median 7, range 5–7).

Although the pilot study was not powered to evaluate efficacy of the intervention, a decrease in depression scores was noted for persons using the *ePST *program as intended, and gains appeared to have been maintained at 1-month follow-up.

### Limitations

The clinical evaluation component of this study was secondary to the technology development, in the amount of time and funding devoted to it, and was primarily intended to establish a methodology to evaluate the program in future studies. As such, the feasibility study has many limitations, including the limited sample size and unique characteristics of the sample (ie, high education and frequent computer use), inequivalent compensation and assessment time points, and lack of an active control condition. Therefore, given these limitations all conclusions must be considered to be highly preliminary, and the *ePST *program should be evaluated with a larger sample that includes an active control condition.

Although we used validated measures wherever possible, none existed for some domains of interest, and we therefore created them. The Cred-Q and AST, which were used for the first time in this study, may have been subject to response set bias. It is notable that most Cred-Q and AST item means are similar to each other, with little variability. Of note are apparently conflicting results to AST questions about preference for using a computer versus being treated by a live clinician: “I would rather do problem-solving treatment with a therapist than with the computer” (mean 5.0, SD 1.4) and “I would rather use a computer to help myself privately than go to a therapist” (mean 5.0, SD 0.8). Therefore, the Cred-Q and AST should be revised if used in subsequent studies to better detect response set. Approaches to designing Likert scales to detect and prevent response set have been advanced by Shulruf et al [41] and Barnette [42].

### Future Directions and Conclusions

Future studies could directly compare the interactive media approach to delivering problem-solving treatment (which is both bandwidth intensive and costly) versus a text-based version that functions using the same logic. This could determine the merit of using interactive media, compared to the cost to produce and deliver it.

Further evaluation of the effectiveness of *ePST *is warranted; however, the question is what the best design for such as study would be. Historically, computer-automated behavioral interventions have generally been compared with a waitlist (as in this study), usual care, or live therapy. However, there are drawbacks to each of these designs. Some treatment is likely to be better than no treatment, making a waitlist control a straw man (although in many clinics patients linger on waitlists for weeks and months, making this a de facto usual care comparison). Usual care comparisons may have more real-world applicability, in which participants are randomly assigned to either use *ePST *or receive treatment as usual. But usual care varies between settings, and even within a single study group may include many interventions, such as receiving medication, intensive therapy, or nothing at all. Therefore, the usual care control is actually an amalgam of multiple types of control groups.

Randomized “John Henry” [43] (man versus machine) trials that compare *ePST *versus live problem-solving treatment clinicians following the same protocol would establish *noninferiority*. This type of data would be most useful for clinics deciding whether to deliver problem-solving treatment via computer or a live clinician (and for which patients); however, many settings do not have the resources to hire or train sufficient numbers of problem-solving treatment clinicians to meet the demand for treatment. A more useful comparison might be randomization to either *ePST *or another automated treatment of depression, now that several exist in the marketplace. Clinics that are unable to hire specialty staff may well be able to provide automated depression treatment, and few head-to-head comparisons of computer-automated depression treatments have been published.

A limitation across all of the above comparisons is that they evaluate the automated treatment as a static entity and preclude the opportunity for its improvement during the trial. The criterion-based development model proposed by Carter [44] is a cyclical model of testing and evaluating to criterion. In this model, originally for the evaluation of self-instructional interactive media programs, an a priori target level of learner performance is established, which signifies an acceptable level of mastery over a skill. Essentially, “*X*% of learners should perform skill *Y *at level *Z*.” The interactive media program is then tested and revised in a spiral development model [45] until the training criteria are met. The criterion-based development model can be applied to the evaluation of self-treatment software, as well, by establishing a meaningful reduction of depression symptoms and functional impairment for users, and testing and revising the program to reach that criterion.

The advantage of the criterion-based development model over randomized trials is that evaluation data are immediately applied to the improvement of the program, maximizing the potential of the program to effect change. Rather than comparing treatment conditions, which may yield statistically, but not clinically, significant differences, evaluation group outcomes are compared with predetermined, objective standards of health and functioning. It is possible to test whether this meaningful goal is met or exceeded. Clearly there is a need for both randomized trial and criterion-based evaluations, and *ePST *should be tested using both models.

Further development of *ePST *could tailor the program to special populations, such as cancer patients, older persons, or adolescents. To increase accessibility, a range of hosts could be provided, to better match the user’s demographics, including his or her language.

Although other computer-automated treatments for depression have previously been developed and shown to be effective, that does not mean work in the field should stop, any more than drug discovery should cease once an effective medication has been found for a disease. *ePST *was designed to overcome some limitations of current computer-automated therapies, such as reliance on text, limited interactivity, and little if any feeling of human connection. In doing so, it presents a model for next-generation interactive, media-based interventions.

## Abbreviations

AST: Assessment of Self-guided Treatment Questionnaire
Cred-Q: Credibility Questionnaire
DSM-IV-TR: Diagnostic and Statistical Manual of Mental Disorders, 4th edition, text revision
ePST: electronic problem-solving treatment
HDI: Hamilton Depression Inventory
HDRS: Hamilton Depression Rating Scale
MINI: Mini-International Neuropsychiatric Interview
NASA: National Aeronautics and Space Administration
PHQ-9: Patient Health Questionnaire-9
PST-PC: problem-solving treatment for primary care
SUS: System Usability Scale

## References

[1] Cartreine JA, Ahern DK, Locke SE (2010). A roadmap to computer-based psychotherapy in the United States. Harv Rev Psychiatry.

[2] Peck DF (2010). The therapist-client relationship, computerized self-help and active therapy ingredients. Clin Psychol Psychother.

[3] Arean P, Hegel M, Vannoy S, Fan MY, Unuzter J (2008). Effectiveness of problem-solving therapy for older, primary care patients with depression: results from the IMPACT project. Gerontologist.

[4] Mynors-Wallis LM, Gath DH, Lloyd-Thomas AR, Tomlinson D (1995). Randomised controlled trial comparing problem solving treatment with amitriptyline and placebo for major depression in primary care. BMJ.

[5] Mynors-Wallis LM, Gath DH, Day A, Baker F (2000). Randomised controlled trial of problem solving treatment, antidepressant medication, and combined treatment for major depression in primary care. BMJ.

[6] Hegel MT, Arean PA (2003). University of Washington, Department of Psychiatry & Behavioral Sciences.

[7] Warmerdam L, van Straten A, Jongsma J, Twisk J, Cuijpers P (2010). Online cognitive behavioral therapy and problem-solving therapy for depressive symptoms: Exploring mechanisms of change. J Behav Ther Exp Psychiatry.

[8] Hegel MT, Barrett JE, Cornell JE, Oxman TE (2002). Predictors of response to problem-solving treatment of depression in primary care. Behav Ther.

[9] Oxman TE, Hegel MT, Hull JG, Dietrich AJ (2008). Problem-solving treatment and coping styles in primary care for minor depression. J Consult Clin Psychol.

[10] Barrett JE, Williams JW, Oxman TE, Katon W, Frank E, Hegel MT, Sullivan M, Schulberg HC (1999). The treatment effectiveness project. A comparison of the effectiveness of paroxetine, problem-solving therapy, and placebo in the treatment of minor depression and dysthymia in primary care patients: background and research plan. Gen Hosp Psychiatry.

[11] Carter JA, Buckey JC, Greenhalgh L, Holland AW, Hegel MT (2005). An interactive media program for managing psychosocial problems on long-duration spaceflights. Aviat Space Environ Med.

[12] Henderson JV (1998). Comprehensive, technology-based clinical education: the “Virtual Practicum”. Int J Psychiatry Med.

[13] Henderson JV, Rossett A (2003). Comprehensive clinical education using “advanced” multimedia: the virtual practicum. Rossett A, editor. Proceedings of World Conference on E-Learning in Corporate, Government, Healthcare, and Higher Education.

[14] Boisot MH (1995). Information Space: A Framework for Learning in Organizations, Institutions, and Culture.

[15] Kolb DA (1984). Experiential Learning: Experience as the Source of Learning and Development.

[16] Schön DA (1987). Educating the Reflective Practitioner: Toward a New Design for Teaching and Learning in the Professions (Higher Education Series).

[17] Henderson JV, Carter JA, Daetwyler C (2010). Interactive Media Laboratory, Dartmouth Medical School.

[18] Henderson JV (2010). Interactive Media Laboratory, Dartmouth Medical School.

[19] Henderson JV (2010). Interactive Media Laboratory, Dartmouth Medical School.

[20] Kroenke K, Spitzer RL (2002). The PHQ-9: a new depression diagnostic and severity measure. Psychiatr Ann.

[21] Fogg BJ (2002). Persuasive Technology: Using Computers to Change What We Think and Do.

[22] Fogg BJ, Swani P, Treinen M, Marshall J, Laraki O, Osipovich A, Varma C, Fang N, Paul J, Rangnekar A, Shon J (2001). What makes websites credible?.

[23] Lengel RH (1983). Managerial Information Processing and Communication Media Source Selection Behavior [doctoral dissertation].

[24] Daft RL, Lengel RH (1984). Information richness, a new approach to managerial behavior and organization design. Res Organ Behav.

[25] Byrne Z, LeMay E (2006). Different media for organizational communication: perceptions of quality and satisfaction. J Bus Psychol.

[26] Nishida I.S. (1992). Failure Analysis in Engineering Applications. Volume 43.

[27] Starbuck WH, Baumard P (2005). Learning from failures: why it may not happen. Long Range Plann.

[28] Hewett TT (1991). Importance of failure analysis for human-computer interface design. Interact Comput.

[29] Sutcliffe A, Rugg G (1998). A taxonomy of error types for failure analysis and risk assessment. Int J Hum Comput Interact.

[30] Price M American Association for the Advancement of Science.

[31] American Psychiatric Association (2000). Diagnostic and Statistical Manual of Mental Disorders: DSM-IV-TR.

[32] Dozois DJ (2003). The psychometric characteristics of the Hamilton Depression Inventory. J Pers Assess.

[33] Sheehan DV, Lecrubier Y, Sheehan KH, Amorim P, Janavs J, Weiller E, Hergueta T, Baker R, Dunbar GC (1998). The Mini-International Neuropsychiatric Interview (M.I.N.I.): the development and validation of a structured diagnostic psychiatric interview for DSM-IV and ICD-10. J Clin Psychiatry.

[34] Reynolds WM, Kobak KA (1995). Reliability and validity of the Hamilton Depression Inventory: a paper-and-pencil version of the Hamilton Depression Rating Scale clinical interview. Psychol Assess.

[35] Hamilton M (1960). A rating scale for depression. J Neurol Neurosurg Psychiatry.

[36] Brooke J, Jordan P. W., Thomas B., Weerdmeester B. A., McClelland I. L. (1996). SUS: a quick and dirty usability scale. Jordan PW, Thomas B, Weerdmeester BA, McClelland IL, editors. Usability Evaluation in Industry.

[37] Bangor A, Kortum PT, Miller JT (2008). An empirical evaluation of the System Usability Scale. Int J Hum Comput Interact.

[38] Fogg LF, Rose RM (1995). Use of personal characteristics in the selection of astronauts. Aviat Space Environ Med.

[39] Borkovec TD, Nau SD (1972). Credibility of analogue therapy rationales. J Behav Ther Exp Psychiatry.

[40] Thornett AM, Mynors-Wallis LM (2002). Credibility of problem-solving therapy and medication for the treatment of depression among primary care patients. Med Sci Monit.

[41] Shulruf B, Hattie J, Dixon R (2008). Factors affecting responses to Likert type questionnaires: introduction of the IMPEXP, a new comprehensive model. Soc Psychol Educ.

[42] Barnette JJ (2000). Effects of stem and Likert response option reversals on survey internal consistency: if you feel the need, there is a better alternative to using those negatively worded stems. Educ Psychol Meas.

[43] Johnson GB (1969). John Henry: Tracking Down a Negro Legend.

[44] Carter JA (2005). The criterion-based development model for media-based self-instructional training programs. Behav Ther.

[45] Boehm B (1988). A spiral model of software development and enhancement. Computer.

